# Outcomes of Rubber Band Ligation in Haemorrhoids Among Outdoor Patients

**DOI:** 10.7759/cureus.29767

**Published:** 2022-09-29

**Authors:** Mukesh Kumar, Vivek Roy, Sanjit Prasad, Pradeep Jaiswal, Nidhi Arun, Krishna Gopal

**Affiliations:** 1 General Surgery, Indira Gandhi Institute of Medical Sciences, Patna, IND; 2 Anaesthesiology, Indira Gandhi Institute of Medical Sciences, Patna, IND

**Keywords:** day case surgery, prolapse, bleeding, 2nd degree and 3rd degree haemorrhoids, rubber band ligation procedure

## Abstract

Background: Out of all anorectal diseases, haemorrhoids are the most common benign disease. Haemorrhoids can be treated by various treatment modalities like medical, surgical, and instrumental. Instrumental treatment comprises rubber band ligation, sclerotherapy, and infrared and laser therapy. Out of these modalities, the rubber band ligation technique is the least invasive with a reduced rate of complications and without the need for hospitalization. Hence, the current study was conducted to evaluate the outcomes with respect to the effectiveness of rubber band ligation in grade II and III internal haemorrhoids along with the magnitude and pattern of post-procedural complications.

Methods: This is a prospective observational study, conducted on a sample of 100 patients who presented to our outdoor patient’s department and were diagnosed with haemorrhoids, either grade II or III. All enrolled study patients having haemorrhoids were banded with rubber band by Barron Ligator (Precise, Canada) with local anaesthetic agent xylocaine jelly in a single session. All patients were followed on the 10th day, 1st month, and 6th month after the procedure to assess symptomatic improvement. The endpoint of this study is to know the effectiveness of rubber band ligation in different clinical parameters such as post-ligation pain or discomfort, the requirement of analgesic, any complication, and time off work.

Results: Out of 100 patients 17 patients had grade II and 83 patients had grade III haemorrhoids. Among them, 89% were symptomatically relieved after rubber band ligation whereas the rest 11% of patients had residual symptoms.

Conclusion: Thus, we conclude that rubber band ligation for grade II and III haemorrhoids is simple, safer, easy-to-perform daycare procedure with lesser requirements of analgesics and without any need for anaesthesia.

## Introduction

Among known anorectal diseases, haemorrhoids are the most common benign problem. It has a high prevalence range from 2.9% to 27.9% worldwide out of which more than 4% are symptomatic haemorrhoids. In clinical practice, haemorrhoids are commonly seen and involve any age group. The male and female both sexes are equally affected [1,2]. In haemorrhoids, symptoms are bleeding per rectum, pain, pruritis, and mass per rectum. Haemorrhoids are seen in different groups of populations from low socioeconomic to affluent society people. Haemorrhoids occur due to weakness and venous engorgement of the normal anal cushions. Various techniques of medical, surgical, and instrumental treatment are available according to the degree of haemorrhoids. In medical treatment like a hot sitz bath, laxatives and flavonoids are useful. Among several surgical techniques, from the less invasive like the arterial ligation using HAL (hemorrhoidal artery ligation) doppler, to the more invasive like the Milligan-Morgan procedure, however, our study is focused on the instrumental technique; the elastic ligation, which is not a surgery. While many nonoperative techniques like rubber band ligation, photocoagulation, sclerotherapy, cryotherapy, and minimally invasive techniques are effective in controlling symptoms in patients’ prospects but they share about recurrences [3,4]. Rubber band ligation is a daycare procedure due to no need for admission and using local anaesthetic agents like xylocaine jelly 2% with less complication and recurrence rate. Grade III haemorrhoids are usually managed by instrumental treatment, sometimes in conjunction with well-followed medical treatment. Surgical treatment is more appropriate for grade IV haemorrhoids. In a worldwide scenario, interno-external haemorrhoids are usually surgically treated (open/closed/minimal invasive). In grade II haemorrhoids the most effective non-invasive procedure is rubber band ligation. In grade III haemorrhoids there are some conflicts between instrumental techniques in conjunction with well-followed medical treatment and only instrumental techniques among surgeons for rubber band ligation procedures. In third-degree haemorrhoids, different scientific study has been published in support of the efficacy of rubber band ligation [5,6]. Currently, it is non-invasive associated with minimal pain, and is regularly being done as a daycare procedure along with faster recovery with no hospital admission. With this background; we hypothesize that undertaking this study to evaluate the outcomes with respect to effectiveness in second-degree to third-degree haemorrhoids for rubber band ligation along with the magnitude and pattern of post-procedural complications at the outdoor of our Department of General Surgery.

## Materials and methods

This study was done in the department of general surgery in Indira Gandhi Institute of Medical Sciences (IGIMS), Patna, on 100 outdoor patients complaining of bleeding per rectum with mass spontaneously reduced; second degree and in which manual reduction of mass required; third degree of hemorrhoids in one year duration period. The study was an observational cross-sectional prospective study on the outcome of rubber band ligation in the second- and third-degree cases of internal haemorrhoids. The study was approved by the Institutional Ethical Committee, IGIMS, Patna (Letter no. 932/IEC/IGIMS/2019, dated 03.07.2019) and registered with the Clinical Trial Registry of India (CTRI).

Haemorrhoids were classified by the Modified Golligher Grading system [7]. Patients who had internal haemorrhoids of grade II, who failed to take medical treatment with the accompanying measure of hygiene of life (physical activity etc.), and grade III of either sex in adult patients 14 years or above were included in this study. Patients who had first-degree haemorrhoids, fourth-degree haemorrhoids, external haemorrhoids, or those who did not show up for follow-up were excluded.

In every patient, after informed and written consent was taken, a detailed history and examination were carried out. In daycare surgery, patients completing the inclusion criteria were subjected to rubber band ligation with aseptic precautions. bowel preparation was given to all patients before the procedure to avoid bowel peristalsis for the first 24 hrs to avoid ligature slippage. In the left lateral sims position, the rubber band ligation procedure was done without anaesthesia. After the local application of xylocaine jelly, the proctoscope was introduced and inserted up to 1-2 cm above the dentate line. On gentle withdrawal of the proctoscope, haemorrhoidal cushions were allowed to appear in the lumen of the proctoscope and after that with negative pressure pulled into the Barron Ligator (Precise, Canada). The tissues were drawn into the tip of the cylindrical portion of the ligator until it was elongated and tightened. After that trigger was released, implementing a Barron rubber o-ring band with an inner diameter of about 1mm around the base of the haemorrhoid. All primary haemorrhoids were ligated in one setting. At the end of the procedure, all patients were kept under observation for 1-2 h to detect any early complications such as bleeding, pain, urinary retention, and vasovagal attack. We advised sitz bath at room temperature, a high fibre rich diet, stool softener, proper anal hygiene, to avoid constipation, and proper counselling regarding early and late complications. Outcome parameters such as post-ligation pain or discomfort, the requirement of an analgesic drug, any complications, and time off work were observed. Patients were followed on the 10th day, 1 month, and on 6 months after post-procedure.

## Results

In this study, most of the patients were in 36 to 45 years of age with 68 (68%) and a minimum group of patients below 25 years (5%) (Table 1).

**Table 1:** Number with the percentage of participants in different age groups

Age (year)	Number of participants	(%)
≤25	5	5
26-35	20	20
36-45	68	68
46-55	7	7
Total	100	100

On the basis of the clinical presentation of patients majority of participant that is 92% had bleeding as the predominant symptom and the next common symptom was prolapse during defecation (81%); other symptoms like pain (27%), irritation(22%) and discharge(18) were found in this study (Table 2).

**Table 2:** Distribution of participants according to clinical presentation

Clinical presentation	Number of participants (N=100)	(%)
Bleeding	92	92
Prolapse	81	81
Pain	27	27
Irritation	22	22
Discharge	18	18

Only 17% of the participants were in 2nd-degree haemorrhoids who underwent rubber band ligation having bleeding and spontaneous reducible mass per rectum. The remaining 83% were in third-degree haemorrhoids with complain of bleeding and mass per rectum and required manual reduction (Table 3).

**Table 3:** Distribution of cases according to the grade of haemorrhoids

Degree	Number of participants	(%)
2^nd^ Degree	17	17
3^rd^ Degree	83	83
Total	100	100

On the basis of the assessment of post-procedure discomfort by the Visual Analog Scale (VAS), 78% had no discomfort, 13% patients had mild discomfort, 9% had moderate discomfort (≥3 days) and none of the patients had a complaint of severe discomfort (Table 4).

**Table 4:** Distribution of cases as per the assessment of post-procedure discomfort by VAS VAS: Visual Analog Scale

Post procedure Discomfort	Number of participants	(%)
VAS 0-2	78	78
VAS 3-4	13	13
VAS 5-7	9	9
VAS 8-10	0	0

On the basis of the requirement of the analgesic drug, 78% of patients required no analgesic, 16% required analgesic for 1-3 days, and 6% required analgesia for more than 3 days (Table 5).

**Table 5:** Distribution of patients according to the duration of analgesic requirement

Pain Relief (uses of analgesic drug)	Number of participants	(%)
No	78	78
1-3 days	16	16
>3 days	6	6
Total	100	100

Most of the participants (79%) resumed their routine work day after the procedure while 7% of participants had joined work 4 days post the procedure. The rest 14% were joined to work within 1 to 3 days (Table 6).

**Table 6:** Distribution of patients according to number of off-days from duty

Leave from Work	Number of participants	(%)
None	79	79
1-3 days	14	14
>4 days	7	7
Total	100	100

Complication after the rubber band ligation is minor and sometimes severe which requires admission. In 22% of cases complains of pain in the anal area and bleeding per rectum in 18% of cases after rubber band ligation. Three patients had urinary retention which required catheterization and no other complication of infection or vasovagal attack was observed (Table 7).

**Table 7:** Distribution of patients with post-procedural complications

Complication	Number of participants	(%)
Pain	22	22
Bleeding per rectum	18	18
Retention of urine	3	3
Infection	0	0
Vasovagal attack	0	0

After a follow-up on the 10th day, one month, and six months of post-procedural rubber band ligation clinical symptoms such as bleeding per rectum, pain, and prolapse of piles mass gradually decreased. Irritation and discharge subsided after the 10th day of post-procedural. Details of parameters observed during the follow-up visit are summarized in Table 8.

**Table 8:** Distribution of cases according to the persistence of symptoms in follow-up

Clinical features	1^st ^day presentation		10th day of post-procedure		1 month of post-procedure		6th month of post-procedure	
	Number	Percentage	Number	Percentage	Number	Percentage	Number	Percentage
Bleeding	92	92	18	18	6	6	4	4
Pain	27	27	7	7	5	5	3	3
Prolapse	81	81	9	9	6	6	4	4
Irritation	22	22	0	0	0	0	0	0
Discharge	18	18	0	0	0	0	0	0

## Discussion

Nowadays a lot of treatment options are present depending upon clinical features and degree of haemorrhoid. While performing haemorrhoidal surgery it has to be safe and no life-threatening condition to be there. Open haemorrhoids surgery, i.e., the Milligan-Morgan technique which was illustrated in 1937 and is still now being widely performed today with certain minor modifications and especially for fourth-degree haemorrhoids and strangulated haemorrhoids. It is widely accepted worldwide, in spite of being associated with post-operative pain. In 1954, Blaisdell described the instrument of rubber band ligation which was further modified by Barron [8]. In 400 patients with 1st degree, 2nd degree, and 3rd degree of haemorrhoids, Barron performed a rubber band ligation procedure while in 750 patients with 2nd degree and 3rd degree of haemorrhoid, El Nakeeb et al. have done same procedure; each study show equal results up to 3rd degree of internal haemorrhoids [9]. Worldwide,in clinical studies of rubber band ligation alone or with comparison to other surgical treatment average success rate of the procedure is approximately 75% or a maximum of 92% has been reported [10]. In this study, the success rate was 89% found which correlates with the above-mentioned studies.

It has been documented in various studies that the repeat requirement of rubber band ligation was 6% to 20%, and 18% of the patients required repeat rubber band ligation in Bayer et al. study while in the same study 2.1% of patients required conventional haemorrhoidal surgery [11]. In this study, still, 11% have symptoms after six months follow up which require conventional haemorrhoidectomy.

In 2.2% of patients who were having complications of bleeding per rectum were reported in trials conducted by Komborozos et al. [12] and Bat et al. [13], bleeding is the most complication after rubber band ligation which cannot be prevented. It is due to local inflammation and fall of the haemorrhoidal nodule. This study shows 6.0% after one month of rubber band ligation procedure and 4% after six months of post-procedure.

Forlini et al. in 2009 advocated the safety of rubber band ligation based on resolutions of presenting symptoms, absence of repeat treatment, satisfaction reported by patients, and time before returning to work [6]. Johansson et al. showed that 6.6-14% of the patients undergoing rubber band ligation required additional treatment, due to the recurrence of symptoms but only 10% of such patients require excisional haemorrhoidectomy. In this study, 11% still have symptoms after six months which require conventional surgery [14].

Savioz et al. reported that symptomatic recurrence was 11.95% after rubber band ligation with 9.2% requiring repeat rubber band ligation or surgery [15]. In this study, we have found that 4% of patients have bleeding per rectum after six months post-procedure.

Lee et al. reviewed 39 studies incorporating 8060 patients undergoing rubber band ligation revealed post-banding complications in the form of severe pain (5.8%), bleeding per rectum (1.7%), infection (0.05%), and 415 patients having mild pain 24 to 48 hours after the post-procedure, which could be relieved through warm sitz baths and oral analgesics [16]. Gehamy and Weakley reported the same results. In this study, 78% of patients do not require any analgesic drug and are relieved by a warm sitz bath while 16% of patients require analgesics for 1-3 days and only 6% of patients take analgesics for more than three days [17]. Hardwick and Durdey did not show any relationship between the number of bands applied and the degree of pain [18]. Moreover, Khubchandani IT in a prospective study compared the results of single, double, and triple hemorrhoidal ligation. In this study, repeated band ligation was in 11% of patients [19].

Bat et al. showed that the complications rate after rubber band ligation was relatively low (4.2%) and most of the complications were minor and self-limiting [13]. In our study, 3% of patients had retention of urine with a minor complication of bleeding and discomfort.

Gagloo et al. in a retrospective analysis of short- and long-term efficacy of rubber band ligation for haemorrhoids observed that at the end of two months, 92% of second-degree and 76% of third-degree patients show no complications [10]. In this study, at the end of 1st month, 17% of patients and 11% of patients have complications after six months which include bleeding, pain, and prolapse.

Walker et al. reported in their study that 25% of patients were associated with postprocedural complications and 20.2% were absent from work. In this study, 79% of patients resumed working the next day after the procedure [20].

This study is conducted at only a single center. Observing the increasing incidence of haemorrhoids, a multicentric study is needed to evaluate the efficacy of rubber band ligation in management of grade II and grade III haemorrhoids. Dietary and life-style modifications are important components of the management of haemorrhoids. We have suggested the required dietary and lifestyle modification to all the patients enrolled, but long-term post-operative lifestyle adjustment was not controlled or followed up in our study. These were a few limitations of our study.

## Conclusions

Haemorrhoids is the most common benign anorectal pathology common among adults and predominantly in male. Rubber band ligation is a daycare instrumental procedure that is convenient for patients and has no need for anaesthesia. It is a relatively effective method of treatment in second-degree and third-degree haemorrhoids in which patients required fewer analgesics, perceived fewer complications, and returned quickly to duties. It is also an emergency procedure, to control active bleeding from haemorrhoids. We found rubber band ligation technique is more effective in second-degree haemorrhoid than in third-degree haemorrhoid, but it helps control bleeding and prolapse and is most widely done for patients who are unfit for surgery or have a co-morbid condition that contraindicates anaesthesia but is not used in thrombosed haemorrhoids.
